# Preoperative and intraoperative tirofiban during endovascular thrombectomy in large vessel occlusion stroke due to large artery atherosclerosis

**DOI:** 10.1111/ene.16419

**Published:** 2024-07-29

**Authors:** Zhiqiang Sun, Shuhan Huang, Wei Li, Yi Yang, Ya Wu, Xue Ma, Ximing Nie, Wangsheng Jin, Chengchun Liu, Xiaoshu Li, Yaning Xu, Jun Dong, Yisi Liao, Binlu Sun, Wenjun Han, Qing Zhao, Huaqiao Chi, Yanjiang Wang, Liping Liu, Meng Zhang

**Affiliations:** ^1^ Department of Neurology Daping Hospital, Third Military Medical University Chongqing China; ^2^ Department of Neurology First Affiliated Hospital of Chongqing Medical University Chongqing China; ^3^ Department of Neurology Beijing Tiantan Hospital, Capital Medical University Beijing China; ^4^ China National Clinical Research Center for Neurological Diseases Beijing China; ^5^ Department of Neurology 985 Hospital of Joint Logistics Support Force Taiyuan China

**Keywords:** endovascular thrombectomy, ischemic stroke, reperfusion, tirofiban

## Abstract

**Background and purpose:**

The aim of this study is to investigate the efficacy and safety of preoperative versus intraoperative tirofiban in patients with large vessel occlusion (LVO) due to large artery atherosclerosis (LAA).

**Methods:**

This is a retrospective multicenter cohort study based on the RESCUE‐RE (Registration Study for Critical Care of Acute Ischemic Stroke After Recanalization) trial enrolling patients with anterior circulation LVO classified as LAA within 24 h of onset. Patients were divided into three groups: preoperative tirofiban (PT), intraoperative tirofiban (IT), and no tirofiban (NT). Propensity score matching (PSM) was used to balance baseline characteristics. The efficacy outcomes included 90‐day functional independence (modified Rankin Scale score = 0–2) and early partial recanalization (EPR; defined as a modified Thrombolysis in Cerebral Infarction score = 1–2a). The safety outcomes included symptomatic intracranial hemorrhage (sICH).

**Results:**

A total of 104 matched triplets were obtained through PSM. Compared with NT, PT increased 90‐day functional independence (60.8% vs. 42.3%, *p* = 0.008) and EPR (42.7% vs. 18.3%, *p* < 0.001) rate, with a tendency to increase the asymptomatic intracranial hemorrhage (aICH) proportion (28.8% vs. 18.3%, *p* = 0.072). Compared with IT, PT had a higher 90‐day functional independence (60.8% vs. 45.2%, *p* = 0.025) and EPR (42.7% vs. 20.2%, *p* = 0.001) rate, with no significant difference in sICH (14.4% vs. 7.7%, *p* = 0.122) and aICH (28.8% vs. 21.2%, *p* = 0.200). Compared with NT, IT had a lower 90‐day mortality rate (9.6% vs. 24.0%, *p* = 0.005).

**Conclusions:**

Tirofiban shows good adjuvant therapy potential in acute ischemic stroke–LVO due to LAA patients. PT is associated with higher rates of EPR and better therapeutic efficacy. In addition, EPR may be a potential way to improve prognosis.

## INTRODUCTION

Large artery atherosclerosis (LAA) is the most common acute ischemic stroke (AIS) subtype worldwide, and the incidence rate can reach up to 33% in Asian populations [1]. One of the main causes of LAA‐related large vessel occlusion (LVO) is the rupture of atherosclerotic plaques based on in situ stenosis, which triggers platelet activation and mediates thrombosis within a short period of time [2]. Mechanical thrombectomy (MT), while removing newly formed thrombus, damages atherosclerotic plaques and vascular endothelium, further activating platelets, increasing the risk of early reocclusion and distal embolism [3, 4, 5]. Therefore, LAA patients have a poorer response to MT and necessitate more intricate recanalization strategies and longer procedure time [6, 7].

Tirofiban is a fast‐acting, highly selective nonpeptide platelet glycoprotein IIb/IIIa receptor inhibitor with a half‐life of approximately 4 h. It can effectively and rapidly block the final pathway of platelet aggregation and subsequent formation of thrombus [8, 9, 10]. Tirofiban is widely used as an antithrombotic rescue drug during endovascular thrombectomy (EVT). Observational studies suggest that it plays a promising role in LAA patients, especially those with severe residual stenosis, angioplasty, or stent implantation, or for the rescue of failed recanalization [11, 12, 13]. In recent years, preoperative tirofiban has attracted more attention. The RESCUE‐BT randomized controlled trial, enrolling 948 patients, conducted in China, showed that the administration of intravenous tirofiban before EVT does not improve the outcome of AIS‐LVO patients [14]. However, the subgroup analysis indicated that it may be effective for patients with LAA. Considering the pharmacological mechanism of tirofiban, we hypothesize that early use may bring considerable benefits for LAA patients. It is unclear whether there are any advantages to preoperative tirofiban versus its intraoperative use in clinical practice. This study aims to evaluate the efficacy and safety of preoperative versus intraoperative tirofiban in LAA patients in the real world.

## METHODS

### Study participants

We analyzed the data from February 2016 to August 2023 in Registration Study for Critical Care of Acute Ischemic Stroke After Recanalization (RESCUE‐RE) [15]. The inclusion criteria were as follows: (i) anterior circulation LVO confirmed by computed tomographic angiography/magnetic resonance angiography; (ii) stroke etiology classified by the TOAST (Trial of Org 10172 in Acute Stroke Treatment) system considered LAA, including intracranial and extracranial arterial atherosclerosis lesions; and (iii) preonset modified Rankin Scale (mRS) score ≤ 2. The main exclusion criteria were as follows: (i) patients with preoperative oral dual antiplatelet drugs, (ii) patients with perioperative use of other intravenous antiplatelet drugs, and (iii) important baseline data missing.

### Standard protocol approval, registration, and patient consent

RESCUE‐RE is a prospective, multicenter cohort study performed at 18 comprehensive stroke centers across China, aiming to evaluate the outcomes of AIS patients with LVO treated with EVT in real‐world clinical practice. The study is registered on the Chinese Clinical Trial Registry (URL: http://www.chictr.org.cn; unique identifier: ChiCTR1900022154), which has been approved by the institutional review board of Beijing Tiantan Hospital, Capital Medical University (approval no. KY2018‐057‐01). All patients or their legal representatives provided written, informed consent to participate in the study.

### Data collection

Extracted data collected during the patient's hospitalization and 3‐month follow‐up included age, sex, history of antiplatelet drug, admission National Institutes of Health Stroke Scale (NIHSS) score, time from last known well to puncture, tirofiban treatment information, neuroimaging data (e.g., Alberta Stroke Program Early Computed Tomography Score [ASPECTS], occlusion site, modified Thrombolysis in Cerebral Infarction [mTICI], and symptomatic intracranial hemorrhage [sICH], defined as any intracranial hemorrhage on post‐EVT cerebral imaging with an increase of ≥4 points on the NIHSS score according to the Second European–Australasian Acute Stroke Study criteria [16]), asymptomatic intracranial hemorrhage (aICH; defined as intracranial hemorrhage that does not have any impact on the prognosis or modification of the patient's treatment, and there is no significant alteration in the patient's neurological condition [17]), stroke etiology, EVT procedure, and 90‐day follow‐up outcomes. The core imaging interpretations of this registered study were conducted by the Imaging Laboratory of Tian Tan Hospital to ensure the consistency of evaluation criteria. Specifically, all images were independently reviewed by two trained readers (Drs. Weibin Gu and Xinyi Hou) blinded to clinical data. If there was a disagreement, a third neuroradiologist would participate in further evaluation.

### EVT procedure

Local or general anesthesia was chosen based on the patient's level of cooperation and medical condition. The interventionists determined the EVT devices and intervention strategies. If there were no contraindications, intravenous thrombolysis (IVT) would be performed before EVT. All patients underwent the first angiography prior to operation and the last angiography before the end of EVT. For residual stenosis >70% of the lesions, balloon dilation and/or stent implantation could be used as remedial treatment.

### Tirofiban

Eligible patients were divided into three groups based on tirofiban use: preoperative tirofiban (PT), intraoperative tirofiban (IT), and no tirofiban (NT). Perioperative use of tirofiban was at the discretion of the interventionists. PT was defined as intravenous administration once the LVO was confirmed on admission imaging and the injection was needed before the beginning of arterial puncture. Tirofiban is usually administrated before EVT when considering the etiological classification of stroke to be LAA, to improve blood flow to the occluded area, prevent thrombus extension, and reduce possible rescue treatment during the procedure. Preoperative diagnosis of LAA is mainly based on the following: (i) the form of onset; (ii) high‐risk factors of atherosclerosis such as diabetes, hypertension, and smoking; (iii) the evidence of intracranial and extracranial artery stenosis in imaging examination; and (iv) no evidence of atrial fibrillation and possible cardiogenic diseases. In the PT group, tirofiban was injected intravenously in a 10‐μg/kg bolus, then maintained at a dose of 0.1 μg/kg per minute. IT was defined as intravenous or arterial infusion after initial angiography, and it should be given before the final angiogram. In the IT group, tirofiban was considered during the EVT procedure when the following applied: (i) to prevent reocclusion of severe residual stenosis, (ii) to prevent distal arterial occlusion, or (iii) emergency stent implantation or balloon angioplasty was performed. The routine practice was arterial or intravenous bolus injection of 10 μg/kg, followed by intravenous continuous infusion at 0.1 μg/kg per minute. NT was defined as tirofiban not being used during the perioperative period. Computed tomography or DynaCT was performed immediately after EVT. If there was no intracranial hemorrhage or severe bleeding, tirofiban was typically switched to oral antiplatelet therapy (loading dose aspirin and clopidogrel/ticagrelor) after 24 h.

### Efficacy and safety outcomes

The primary efficacy outcome was functional independence (mRS = 0–2) at 90 days. The secondary efficacy outcomes included 90‐day mRS distribution and mortality, successful recanalization at final angiogram (mTICI = 2b–3), and time from puncture to recanalization (PTR). In addition, we also explored the effect of preoperative tirofiban on the blood flow state on initial angiography, including early partial recanalization (EPR; defined as an mTICI score of 1–2a on initial angiogram) and early recanalization (ER; defined as an mTICI score of 2b–3 on initial angiogram [18]). The safety outcomes contained sICH and aICH within 48 h.

### Statistical analysis

Descriptive statistics were utilized for summarizing demographic factors, medical history, and baseline characteristics variables, both continuous and categorical. Counts and percentages were reported for categorical variables, and mean (SD) or median (interquartile range [IQR]) was reported for continuous variables. To evaluate the normality of distributions, the Shapiro–Wilk test and histograms were utilized. Chi‐squared or Fisher exact test were used for categorical variables. Mann–Whitney *U*‐test or Student *t*‐test was used for continuous variables.

We used propensity score matching (PSM) to balance the baseline clinical preoperative variables and potential confounders. Patients in the three groups were matched at a 1:1:1 ratio using the TriMatch package (v0.9.9) of the R program. PSM was applied by nearest‐neighbor matching with a caliper of the distance of 0.1. After PSM, baseline characteristics and outcomes of the three groups were compared. Pairwise comparisons of the three groups were conducted with Bonferroni post hoc tests. IBM SPSS Statistics (v27.0) and R (R Foundation for Statistical Computing; v4.3.1) were used for all statistical analyses.

## RESULTS

### Population

**FIGURE 1:**
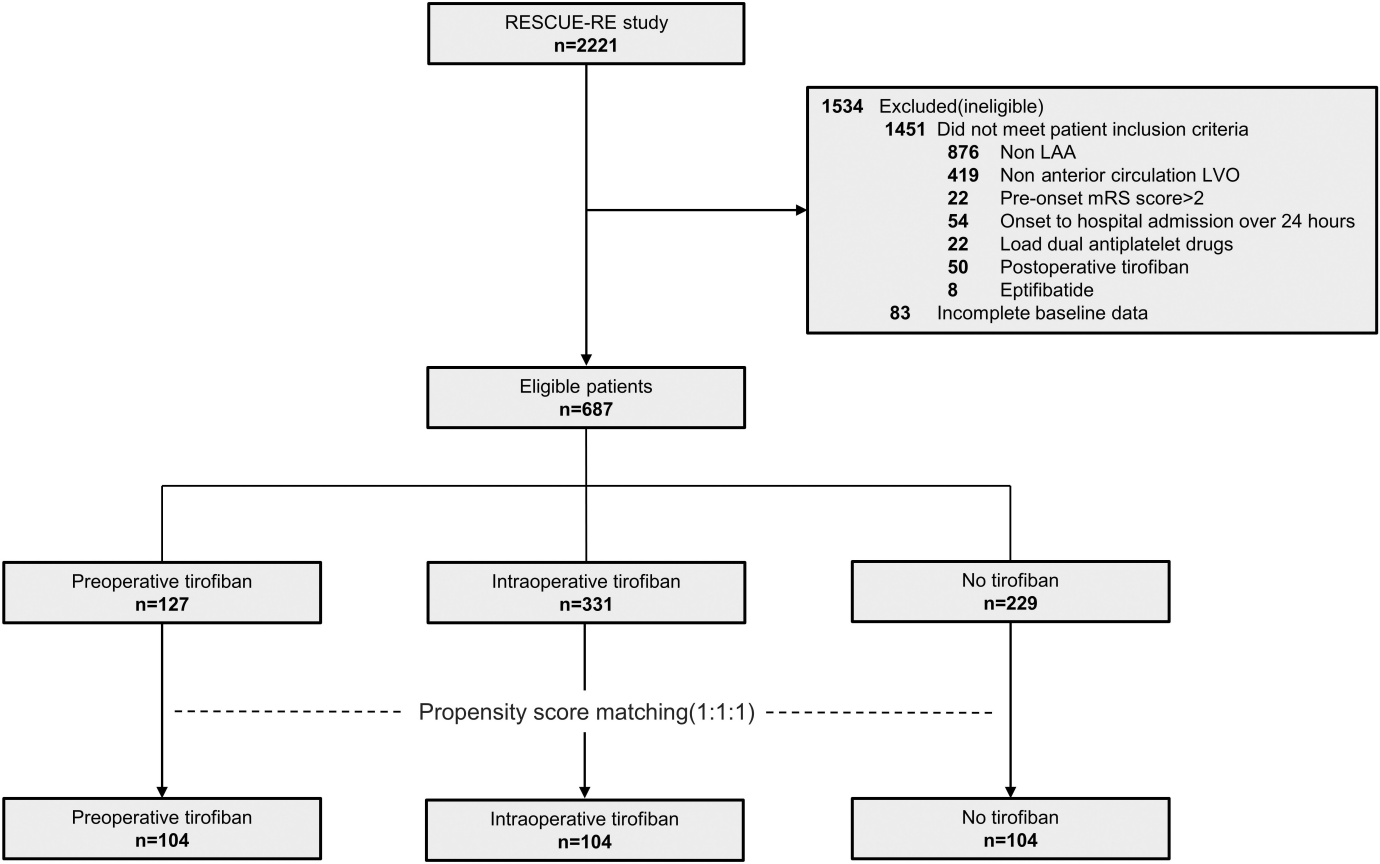
Flowchart. LAA, large artery atherosclerosis; LVO, large vessel occlusion; mRS, modified Rankin scale; RESCUE‐RE, Registration Study for Critical Care of Acute Ischemic Stroke After Recanalization.

A total of 1534 patients were excluded from 2221 in the RESCUE‐RE study. The selection process is detailed in Figure 1. Finally, we enrolled 127 patients in the PT group, 331 patients in the IT group, and 229 patients in the NT group. Among all patients, 508 (73.9%) were male, the median age was 62 years (IQR = 53–70), the median admission NIHSS score was 13 (IQR = 9–17), the median ASPECTS was 8 (IQR = 7–10), and 120 (17.5%) patients had ipsilateral internal carotid artery occlusion.

### Propensity score matching

Baseline characteristics in the different tirofiban groups are summarized in Table 1. There were statistical differences between the three groups in age, transient ischemic attack/stroke, atrial fibrillation, middle cerebral artery, IVT, last known well to puncture time, heparin, and general anesthesia. To achieve a good balance, all the variables were used as covariates for PSM, which resulted in 104 matched triplets with balanced covariates (20 cases matched more than once in the IT group, 31 cases matched more than once in the PT group, 35 cases matched more than once in the NT group). After PSM, there were no significant differences in baseline characteristics among the three groups (*p* > 0.05; Table 2). Two patients (0.64%) were lost to 90‐day follow‐up.

**TABLE 1:** Baseline characteristic of different treatment groups before propensity score matching.

Baseline characteristic	Preoperative tirofiban, *n* = 127	Intraoperative tirofiban, *n* = 331	No tirofiban, *n* = 229	*p*
Age, years, median (IQR)	66 (57–72)	62 (52–69)	62 (53–70)	0.027*
Male	92 (72.4)	252 (76.1)	164 (71.6)	0.446
Smoking	66 (50.2)	191 (57.7)	116 (50.7)	0.218
Drink	52 (40.9)	166 (50.2)	111 (48.5)	0.206
Medical history				
Hypertension	83 (65.4)	220 (66.5)	143 (62.4)	0.615
Diabetes	33 (26.0)	72 (21.8)	48 (21.0)	0.525
TIA/stroke	16 (12.6)	74 (22.4)	57 (24.9)	0.021*
Atrial fibrillation	2 (1.6)	16 (4.8)	22 (9.6)	0.005*
Antiplatelet drug	24 (18.9)	61 (18.4)	54 (23.6)	0.301
Anticoagulant	2 (1.6)	8 (2.4)	8 (3.5)	0.570
Occlusion site				
MCA	88 (69.3)	266 (80.4)	180 (78.6)	0.036*
ICA	49 (38.6)	113 (34.1)	87 (38.0)	0.539
Ipsilateral internal carotid artery occlusion	23 (18.1)	57 (17.2)	40 (17.5)	0.975
Workflow				
NIHSS score, median (IQR)	12 (7–17)	13 (9–17)	14 (11–16)	0.052
ASPECTS, median (IQR)	9 (7–10)	8 (7–10)	8 (7–9)	0.079
IVT	12 (9.4)	87 (26.3)	83 (36.2)	<0.001*
LKW to puncture, min, median (IQR)	628 (415–934)	452 (318–730)	415 (291–660)	<0.001*
Heparin	73 (57.5)	69 (20.8)	64 (27.9)	<0.001*
General anesthesia	9 (7.1)	121 (36.6)	123 (53.7)	<0.001*
Stent implantation	40 (31.5)	106 (32.0)	66 (28.8)	0.712

*Note:* Data are presented as *n* (%) unless otherwise indicated.

Abbreviations: ASPECTS, Alberta Stroke Program Early Computed Tomography Score; ICA, internal carotid artery; IQR, interquartile range; IVT, intravenous thrombolysis; LKW, last known well; MCA, middle cerebral artery; NIHSS, National Institutes of Health Stroke Scale; TIA, transient ischemic attack.

* *p* < 0.05.

**TABLE 2:** Baseline characteristic of different treatment groups after propensity score matching.

Baseline characteristic	Preoperative tirofiban, *n* = 104	Intraoperative tirofiban, *n* = 104	No tirofiban, *n* = 104	*p*
Age, years, median (IQR)	62 (55–71)	61 (53–70)	62 (56–70)	0.699
Male	70 (67.3)	78 (75.0)	75 (72.1)	0.463
Smoking	53 (51.0)	49 (47.1)	63 (60.6)	0.134
Drink	45 (43.3)	42 (40.2)	54 (51.9)	0.220
Medical history				
Hypertension	66 (63.5)	72 (69.2)	73 (70.2)	0.533
Diabetes	29 (27.9)	26 (25.0)	22 (21.2)	0.528
TIA/stroke	15 (14.4)	25 (24.0)	27 (26.0)	0.095
Atrial fibrillation	3 (2.9)	1 (1.0)	1 (1.0)	0.625
Antiplatelet drug	18 (17.3)	16 (15.4)	22 (21.2)	0.544
Anticoagulant	4 (3.8)	3 (2.9)	5 (4.8)	0.932
Occlusion site				
MCA	83 (79.8)	84 (80.8)	82 (78.8)	0.942
ICA	38 (36.5)	36 (34.6)	39 (37.5)	0.907
Ipsilateral internal carotid artery occlusion	19 (18.3)	18 (17.3)	19 (18.3)	0.978
Workflow				
NIHSS score, median (IQR)	13 (8–19)	13 (9–16)	13 (10–18)	0.897
ASPECTS, median (IQR)	8 (7–9)	8 (7–10)	9 (7–10)	0.527
IVT	18 (17.3)	15 (14.4)	18 (17.3)	0.810
LKW to puncture, min, median (IQR)	539 (389–762)	563 (333–844)	444 (315–936)	0.197
Heparin	38 (36.5)	36 (34.6)	36 (34.6)	0.945
General anesthesia	17 (16.3)	16 (15.4)	14 (13.5)	0.839
Stent implantation	37 (35.6)	28 (26.9)	30 (28.8)	0.363

*Note:* Data are presented as *n* (%) unless otherwise indicated.

Abbreviations: ASPECTS, Alberta Stroke Program Early Computed Tomography Score; ICA, internal carotid artery; IQR, interquartile range; IVT, intravenous thrombolysis; LKW, last known well; MCA, middle cerebral artery; NIHSS, National Institutes of Health Stroke Scale; TIA, Transient ischemic attack.

### PT versus NT

**FIGURE 2:**
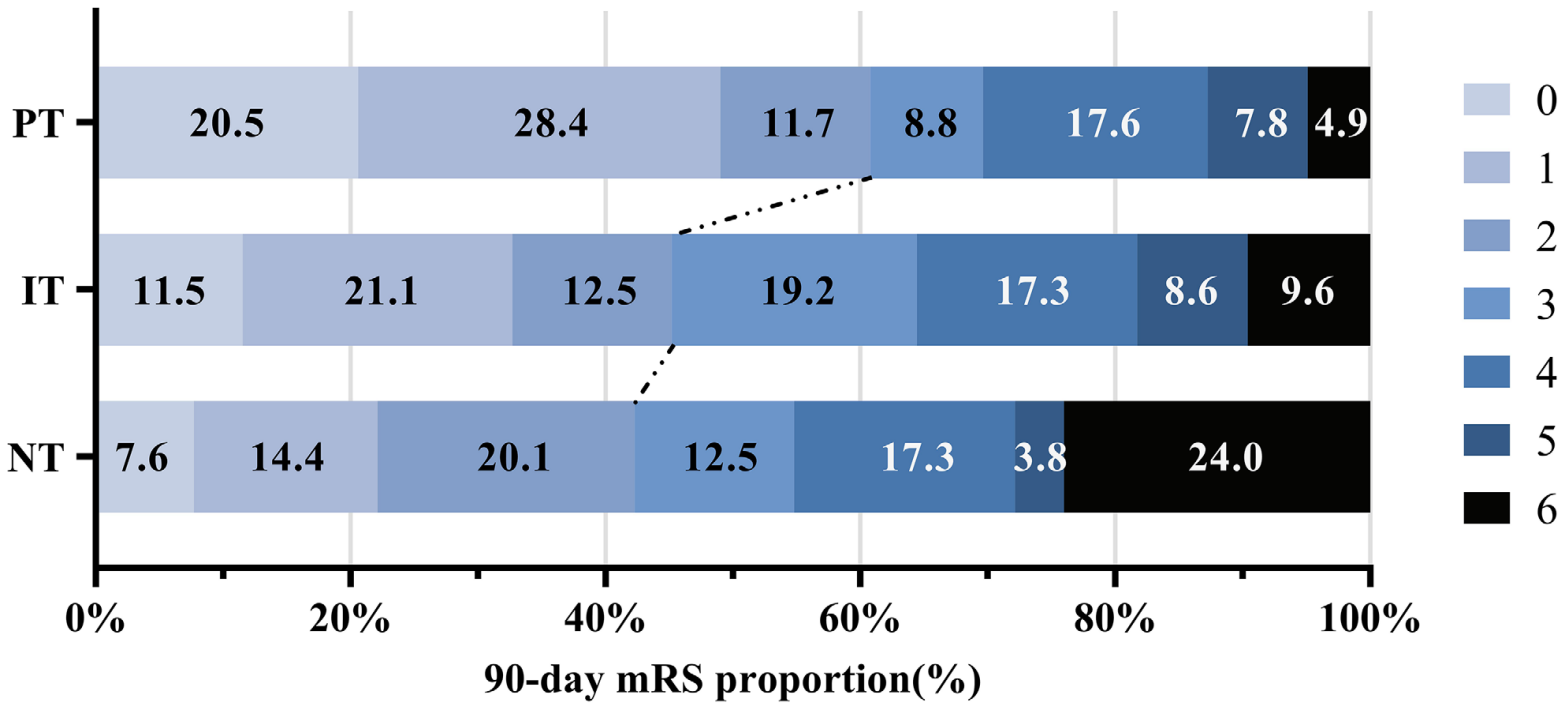
Ninety‐day modified Rankin Scale (mRS) score distribution among the three groups. IT, intraoperative tirofiban; NT, no tirofiban; PT, preoperative tirofiban.

**FIGURE 3:**
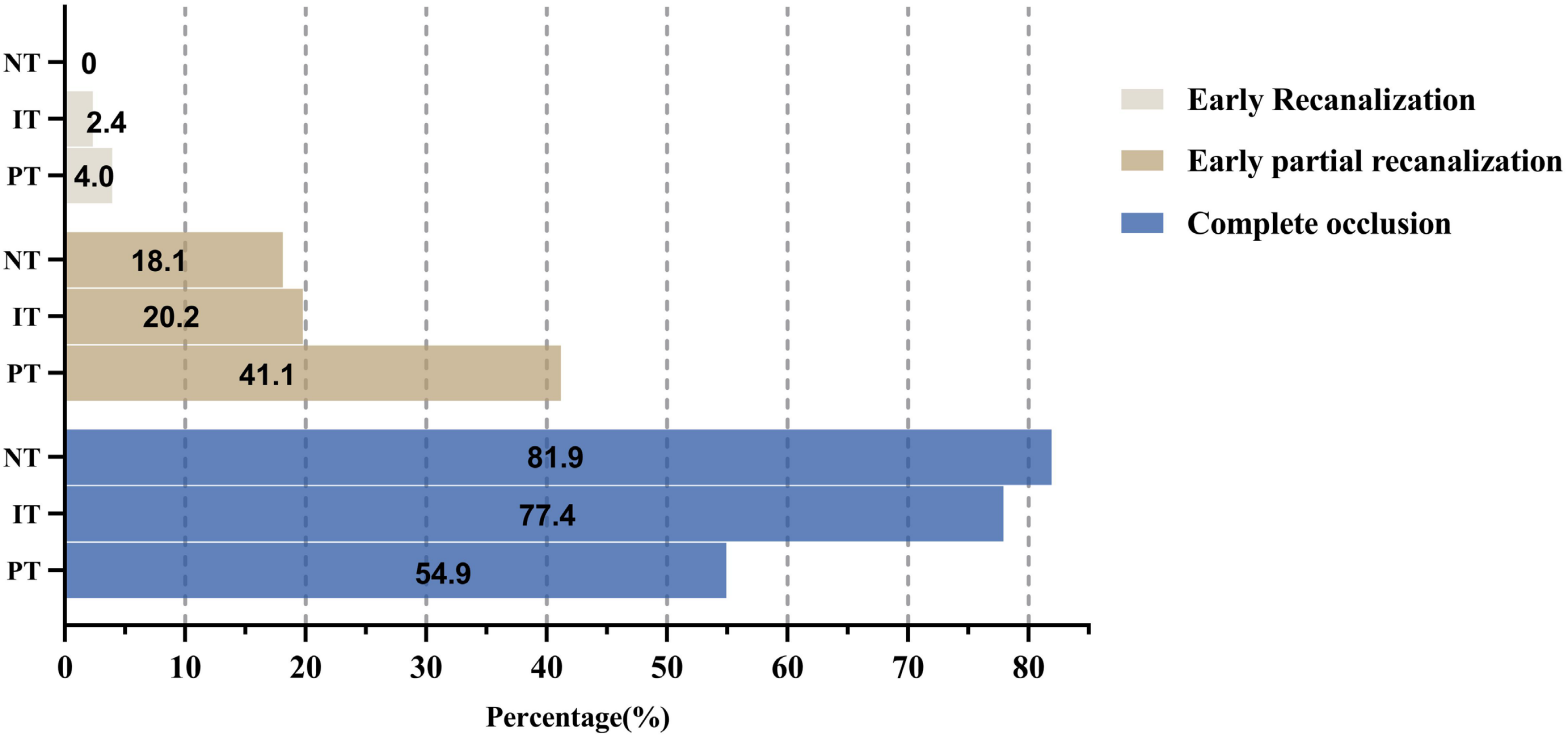
Blood flow status on initial angiography after propensity score matching. Complete occlusion is defined as modified Thrombolysis in Cerebral Infarction = 0. IT, intraoperative tirofiban; NT, no tirofiban; PT, preoperative tirofiban.

The outcomes of the three groups after PSM are shown in Tables 3 and 4. The PT group had a higher rate of functional independence (62 [60.8%] vs. 44 [42.3%], *p* = 0.008), a better mRS score distribution (2 [1–4] vs. 3 [2–5], *p* < 0.001), and a lower proportion of all‐cause mortality (5 [4.8%] vs. 25 [24.0%], *p* < 0.001) at 90 days (Figure 2). For safety outcomes, the sICH incidence in the two groups was similar, at 15 (14.4%) and 17 (16.3%), respectively. The proportion of aICH in the PT group tended to be higher (30 [28.8%] vs. 19 [18.3%], *p* = 0.072), but the difference was not statistically significant. In addition, PT had a higher rate of EPR (41 [42.7%] vs. 17 [18.1%], *p* < 0.001; Figure 3) and successful recanalization (92 [88.5%] vs. 78 [75.0%], *p* = 0.012) and a shorter PTR time (60 [39 to 105] vs. 77 [52 to 137], *p* = 0.025).

**TABLE 3:** Primary and secondary outcomes after propensity score matching.

Outcome	Preoperative tirofiban, *n* = 104	Intraoperative tirofiban, *n* = 104	No tirofiban, *n* = 104	*p*
Efficacy outcomes				
Functional independence at 90 days^a^	62 (60.8)	47 (45.2)	44 (42.3)	0.017*
mRS at 90 days, median (IQR)^a^	2 (1–4)	3 (1–4)	3 (2–5)	<0.001*
Mortality at 90 days^a^	5 (4.8)	10 (9.6)	25 (24.0)	<0.001*
ER^b^	4 (4.2)	2 (2.4)	0	0.123
EPR^b^	41 (42.7)	17 (20.2)	17 (18.1)	<0.001*
Successful recanalization	92 (88.5)	84 (80.8)	78 (75.0)	0.044*
PTR time, min, median (IQR)	60 (39–105)	84 (46–136)	77 (52–137)	0.052
Safety outcomes				
sICH	15 (14.4)	8 (7.7)	17 (16.3)	0.146
aICH	30 (28.8)	22 (21.2)	19 (18.3)	0.171

*Note:* Data are presented as *n* (%) unless otherwise indicated.

Abbreviations: aICH, asymptomatic intracranial hemorrhage; EPR, early partial recanalization; ER, early recanalization; IQR, interquartile range; mRS, modified Rankin Scale; PTR, puncture to recanalization; sICH, symptomatic intracranial hemorrhage.

^a^ Missing 2 patients in preoperative tirofiban group.

^b^ Missing 10 patients in no tirofiban group, missing 8 patients in preoperative tirofiban group, and missing 20 patients in intraoperative tirofiban group.

* *p* < 0.05.

**TABLE 4:** Pairwise comparison of outcomes after propensity score matching.

Outcome	PT versus NT	IT versus NT	PT versus IT
Efficacy outcomes			
Functional independence at 90 days^a^	0.008*	0.675	0.025*
mRS at 90 days^a^	<0.001*	0.070	0.027*
Mortality at 90 days^a^	<0.001*	0.005*	0.193
ER^b^	0.121	0.221	0.686
EPR^b^	<0.001*	0.715	0.001*
Successful recanalization	0.012*	0.316	0.124
PTR time	0.025*	0.963	0.054
Safety outcomes			
sICH	0.701	0.055	0.122
aICH	0.072	0.601	0.200

Abbreviations: aICH, asymptomatic intracranial hemorrhage; EPR, early partial recanalization; ER, early recanalization; IQR, interquartile range; IT, intraoperative tirofiban; mRS, modified Rankin Scale; NT, no tirofiban; PT, preoperative tirofiban; PTR, puncture to recanalization; sICH, symptomatic intracranial hemorrhage.

^a^ Missing 2 patients in PT group.

^b^ Missing 10 patients in NT group, missing 8 patients in PT group, and missing 20 patients in IT group.

* *p* < 0.05.

### IT versus NT

In the comparison after PSM, there was no statistical difference in functional independence between the two groups (47 [45.2%] vs. 44 [42.3%], *p* = 0.675). The IT group had a lower rate of mortality at 90 days (10 [9.6%] vs. 25 [24.0%], *p* = 0.005). In addition, patients in the IT group had a tendency to achieve better 90‐day mRS scores (3 [1–4] vs. 3 [2–5], *p* = 0.070; Figure 2) and a lower rate of sICH (8 [7.7%] vs. 17 [16.3], *p* = 0.055). There was no significant difference in aICH, ER, EPR, successful recanalization, and PTR.

### PT versus IT

After PSM, patients in the PT group achieved a higher percentage of functional independence (62 [60.8%] vs. 47 [45.2%], *p* = 0.025) and better mRS score distribution (2 [1–4] vs. 3 [1–4], *p* = 0.027) at 90 days. Patients in the PT group tended to have shorter PTR time (60 [39–105] vs. 84 [46–136], *p* = 0.054; Figure 2). In addition, there was a significant difference in EPR between the two groups (*p* = 0.001), at 41 (42.7%) and 17 (20.2%), respectively (Figure 3). In comparison between sICH and aICH, the difference is not significant.

## DISCUSSION

This study found that whether compared with IT or NT, prophylactic tirofiban before EVT is associated with better outcomes, but with a potential to increase the risk of aICH. In addition, preoperative tirofiban had a higher EPR rate.

There is currently no consensus on whether the application of tirofiban during the perioperative period of EVT will increase intracranial hemorrhage [11, 12, 19, 20], which may depend on stroke etiology, administration route, dosage, duration of treatment, et cetera. We found that PT had a tendency to increase the risk of aICH when compared to NT, but there was no statistical difference when compared to IT. In the study by Sang et al. [21], the incidence of aICH was only numerically higher in 29.1% in the tirofiban group, compared to 23.2% in the placebo group. In our study, the rates were 28.8% and 18.3%, respectively. One possible explanation is that the recanalization rate was lower in the NT group in our study, which was only 75.0%. In general, patients with successful recanalization may be more likely to develop intracranial hemorrhage compared to those without [22]. A higher reperfusion rate in the PT group may also lead to a higher rate of intracranial hemorrhage. Zhang et al. [23] found that IT after IVT did not increase the risk of intracranial hemorrhage. However, preoperative tirofiban will further advance the usage timing, and earlier use may potentially amplify this risk. Interestingly, in comparison with IT, we did not observe a significant difference in intracranial hemorrhage. This may be attributed to the remedial use of tirofiban during EVT and a higher recanalization rate in the IT group. In addition, the incidence of aICH in our cohort was similar to that of the recent randomized clinical trial study RESCUE‐BT. Considering the improvement of prognosis, the potentially increased aICH brought by PT could be acceptable to some extent. However, concerns about intracranial hemorrhage persist, especially in patients who received IVT previously.

The different functional outcomes in the three groups could relate to how tirofiban affects EPR at the occluded site before EVT and the efficiency of endovascular procedures. Tsao et al. [24] found that tirofiban could partially reverse the platelet‐rich fresh thrombus at early stages. However, in our study, only a few patients experienced ER, suggesting that tirofiban prior to EVT cannot completely remove the thrombus. Interestingly, the EPR rate was higher in the PT group. It seems that tirofiban could reduce thrombus load, increase thrombus permeability, and lead to partial blood flow [25]. Similar effects have been reported in patients with acute coronary syndrome (ACS) [26, 27]. Although the pathogenesis in patients with ACS is similar to that of AIS‐LVO due to LAA [28], the benefits of partial reperfusion in AIS are still controversial because of the considerable heterogeneity of heart and brain tissue. Two studies found that partial reperfusion after IVT was associated with better clinical prognosis [29, 30], but Checkouri et al. [18] came to the opposite conclusion. The reasons for the difference may be due to varying proportions of LAA. In the latter study, LAA accounted for only 15% of all patients. Compared to other types of AIS‐LVO, patients with LAA usually have better collateral status and a smaller ischemic core [31]. Residual perfusion is one of the main determinants of stroke prognosis. Partial forward blood flow, combined with collateral flow, could increase the distal residual reperfusion and slow down the rate of the core growth [32], which may be one of the reasons why EPR could improve the prognosis.

**FIGURE 4:**
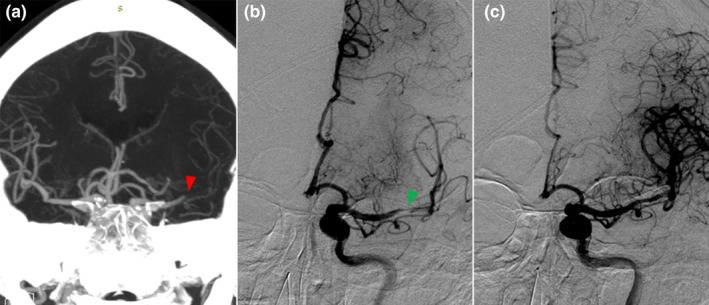
An example of early partial recanalization. (a) Admission computed tomographic angiography indicated complete occlusion of the M1 segment of the left middle cerebral artery (red arrowhead). (b) Tirofiban was administrated before endovascular thrombectomy. The modified Thrombolysis in Cerebral Infarction (mTICI) grade was 2a on initial angiography, indicating early partial recanalization (green arrowhead). (c) After one‐time mechanical thrombectomy and balloon dilation, successful recanalization was achieved, with an mTICI grade of 3 at the final angiography.

Changes in thrombus characteristics and EPR may also facilitate thrombectomy procedures, allowing devices to pass through more easily (typical EPR and EVT procedures are shown in Figure 4). Previous studies have found that tirofiban can reduce the occurrence of intraoperative reocclusion via its continuous platelet aggregation inhibition, which could avert additional passes [4, 13, 21]. These effects were reflected in the shortening of PTR time and the increase of the recanalization rate in our results. A previous meta‐analysis suggested that preoperative tirofiban increased the rate of recanalization compared to rescue use [33], but the difference was not significant in our study. This may be due to the rapid development of thrombectomy devices and technology and the difference in sample size. In our cohort, the better prognosis of PT compared with IT may be attributed to the combined effect of EPR and the improvement of EVT efficiency. This is one of the potential advantages of prophylactic use of tirofiban before EVT as compared to remedial use.

One of the strengths of our study is that on the basis of comparing different timing of administration of tirofiban in AIS‐LVO patients, we investigated the relationship between PT and EPR, and as far as we know, this has not been mentioned in previous research. It provides a new perspective for tirofiban administration before EVT, which awaits further research in the future. For other types of AIS patients, the efficacy and safety of tirofiban still need additional exploration [34, 35, 36, 37].

## LIMITATIONS

Our study had several limitations. First, it was a retrospective study with a small sample size, and although PSM was performed to correct for baseline differences, it could not fully adjust for all confounding factors. Second, tirofiban was used as a rescue treatment after thrombectomy failures in the IT group, which might have caused potential selection bias in some cases. Third, our exploration of EPR is preliminary. Although several studies have found that EPR after IVT significantly improves patient prognosis, whether this applies equally to patients receiving tirofiban has not been confirmed, which needs further research. Fourth, although our study suggests the greater benefit of preoperative tirofiban in LAA patients, it remains challenging for neurologists to determine the etiology of AIS‐LVO stroke early before EVT. Fifth, due to the multicenter nature of this study, there may be high heterogeneity in EVT procedures and perioperative management among different centers. The possibility of center differences influencing some of these results should not be discounted. Sixth, studies find that arterial/intravenous tirofiban may affect patient outcomes, but the conclusions are inconsistent [38, 39]. Due to the limitations of our data, we cannot compare different routes of administration. Considering the above limitations, our findings should be interpreted with caution.

## CONCLUSIONS

Tirofiban could be a potential periprocedural adjuvant therapy for AIS‐LVO due to LAA patients. PT is associated with higher rates of EPR and better therapeutic efficacy. In addition, EPR may be a potential way to improve prognosis. Further randomized controlled trials are needed in the future.

## AUTHOR CONTRIBUTIONS

**Zhiqiang Sun:** Writing – original draft; writing – review and editing; methodology; software; formal analysis; data curation; project administration; validation; visualization; conceptualization. **Shuhan Huang:** Writing – review and editing; methodology; software; project administration; data curation; supervision; writing – original draft; conceptualization. **Wei Li:** Investigation; funding acquisition; writing – review and editing; project administration; supervision; conceptualization. **Yi Yang:** Software; visualization. **Ya Wu:** Funding acquisition; validation; formal analysis. **Xue Ma:** Resources; data curation. **Ximing Nie:** Writing – review and editing; formal analysis. **Wangsheng Jin:** Methodology; writing – review and editing. **Chengchun Liu:** Validation; resources. **Xiaoshu Li:** Validation; software. **Yaning Xu:** Software; writing – original draft; formal analysis. **Jun Dong:** Validation; data curation. **Yisi Liao:** Validation. **Binlu Sun:** Visualization. **Wenjun Han:** Software. **Qing Zhao:** Data curation. **Huaqiao Chi:** Data curation. **Yanjiang Wang:** Project administration. **Liping Liu:** Conceptualization; investigation; funding acquisition; writing – review and editing; supervision; resources; project administration. **Meng Zhang:** Conceptualization; investigation; funding acquisition; writing – review and editing; supervision; resources; project administration.

## FUNDING INFORMATION

This study was supported by the National Natural Science Foundation of China (82071322).

## CONFLICT OF INTEREST STATEMENT

None of the authors has any conflict of interest to disclose.

## Data Availability

Anonymized data related to this study are available from the corresponding author on reasonable request.
